# Influenza and Alcohol-Based Handrub: the Danger of Ignoring Clinical Relevance

**DOI:** 10.1128/mSphere.00719-19

**Published:** 2019-11-27

**Authors:** Alexandra Peters, Didier Pittet

**Affiliations:** a Infection Control Program and WHO Collaborating Center on Patient Safety, University of Geneva Hospitals and Faculty of Medicine, Geneva, Switzerland; National Institute of Allergy and Infectious Diseases

**Keywords:** ABHR, IPC, alcohol-based handrub, hand hygiene, health care workers, infection prevention, influenza

## LETTER

We read the article “Situations leading to reduced effectiveness of current hand hygiene against infectious mucus from influenza virus-infected patients” by Hirose et al. (1) with interest. Unfortunately, although some of the experiments regarding the physical properties of mucus are well performed, the study itself has no clinical relevance, and there are major flaws in its design. The conclusion that current hand hygiene measures are ineffective in preventing what the authors call “contact infection” is inaccurate (and not a term used in infection prevention and control). With the annual flu season rapidly approaching and the article being reported on by a number of mainstream news outlets, such misinformation may have major implications for health care workers’ (HCWs) compliance with hand hygiene and therefore impact patient safety.

The authors show a lack of understanding of clinical practice, current hand hygiene recommendations, and the current literature. Since 2006, when the World Health Organization (WHO) published their draft guidelines on hand hygiene, they have clearly recommend that HCWs use gloves if they anticipate coming into contact with body fluids (2). After the gloves are removed, there should be no physical soil on their hands, at which point they are recommended to perform hand hygiene with alcohol-based handrub (ABHR). If for some reason, the HCW does end up with mucus or other body fluids on their unprotected hands, both the CDC, Atlanta, GA, and WHO recommend physically soiled hands to be washed with soap and water in order to physically remove the contaminating material (3, 4).

Furthermore, the *in vivo* experiment performed did not reflect clinical conditions at all. In order to be effective, ABHR must be applied and rubbed (5). Although the authors spoke of “antiseptic hand rubbing,” hands were not rubbed in their clinical test. Thus, the test used was not consistent with current laboratory practices. If handwashing and hand rubbing were tested using the same (CDC and WHO recommended) technique, the author’s results would no doubt have been quite different. The authors compared placing ABHR solution on fingertips inoculated with influenza A virus (IAV) in mucus with rubbing the volunteers’ hands under running water (without soap) and concluded that hand washing is superior. By this logic, one could also argue that the paper supports washing hands without soap to effectively combat IAV.

The authors’ call for further study so that “more effective ABHR methods can be proposed” is not true. There is no issue with the current methods and recommendations, only with their perceived indications of when to use ABHR and when to wash with (soap and) water. The authors assert that “EBDs [ethanol-based disinfectants] are promoted based on the premise that they inactivate IAV in infectious mucus.” This is false. ABHR is promoted on the premise that it is faster, microbiologically superior in most scenarios for killing pathogens, and better tolerated by skin than handwashing. We strongly recommend to the authors to carefully review the references they cite in their paper.

ABHR is extremely efficacious against the flu—even at lower concentrations, as shown in the paper by Hirose et al. (1)—and remains clinically effective against all other non-spore-forming organisms, including nonenveloped viruses (3). No changes to current infection prevention and control (IPC) practices should be implemented.
